# Pembrolizumab activity in patients with Fanconi anemia repair pathway competent and deficient tumors

**DOI:** 10.1186/s40364-022-00386-0

**Published:** 2022-06-03

**Authors:** Miguel A. Villalona-Calero, John P. Diaz, Wenrui Duan, Zuanel Diaz, Eric D. Schroeder, Santiago Aparo, Troy Gatcliffe, Federico Albrecht, Siddhartha Venkatappa, Victor Guardiola, Sara Garrido, Muni Rubens, Fernando DeZarraga, Hao Vuong

**Affiliations:** 1 City of Hope National Medical Center, Duarte, California USA; 2 Miami Cancer Institute at Baptist Health South Florida, Miami, Florida USA; 3 Departments of Human & Molecular Genetics, Herbert Wertheim College of Medicine, at the Florida International University, Miami, Florida USA; 4 Radiology Associates of South Florida, Miami, Florida USA

**Keywords:** FancD2, Homologous recombination, Immune checkpoint inhibitor, Biomarkers, DNA repair, Fanconi

## Abstract

**Background:**

Given the observed antitumor activity of immune-checkpoint-inhibitors in patients with mismatch-repair deficient (MSI-H) tumors, we hypothesized that deficiency in homologous-recombination-repair (HRR) can also influence susceptibility.

**Methods:**

Patients with disease progression on standard of care and for whom pembrolizumab had no FDA approved indication received pembrolizumab. Patients with MSI-H tumors were excluded. Objectives included immune-related objective response rate (iORR), progression-free survival (PFS) and 20-weeks-PFS. Pembrolizumab was given every 3 weeks and scans performed every six. We evaluated a triple-stain (FANCD2foci/DAPI/Ki67) functional assay of the Fanconi Anemia (FA) pathway: FATSI, in treated patients’ archived tumors. The two-stage sample size of 20/39 patients evaluated an expected iORR≥20% in the whole population vs. the null hypothesis of an iORR≤5%, based on an assumed iORR≥40% in patients with functional FA deficiency, and < 10% in patients with intact HRR. An expansion cohort of MSI stable endometrial cancer (MS-EC) followed. Exploratory stool microbiome analyses in selected patients were performed.

**Results:**

Fifty-two patients (45F,7M;50-evaluable) were enrolled. For the 39 in the two-stage cohort, response evaluation showed 2CR,5PR,11SD,21PD (iORR-18%). FATSI tumor analyses showed 29 competent (+) and 10 deficient (−). 2PR,9SD,17PD,1NE occurred among the FATSI+ (iORR-7%) and 2CR,3PR,2SD,3PD among the FATSI(−) patients (iORR-50%). mPFS and 20w-PFS were 43 days and 21% in FATSI+, versus 202 days and 70% in FATSI(−) patients. One PR occurred in the MS-EC expansion cohort.

**Conclusions:**

Pembrolizumab has meaningful antitumor activity in malignancies with no current FDA approved indications and FA functional deficiency. The results support further evaluation of FATSI as a discriminatory biomarker for population-selected studies.

**Supplementary Information:**

The online version contains supplementary material available at 10.1186/s40364-022-00386-0.

## Introduction

Among the barriers to the generalized applicability of immune checkpoint inhibition as a therapeutic strategy is the identification of patients who will derive the most benefit. Le et al. reported a seminal phase 2 study that eventually led to the Food and Drug Administration’s (FDA) approval of pembrolizumab for the treatment of patients with advanced mismatch repair deficient tumors (MMR-d) [1, 2]. The investigators studied 41 patients with progressive metastatic carcinoma [1]. For patients with MMR-d colorectal cancer, the immune-related objective response rate (iORR) and 20-weeks immune-related progression-free survival (PFS) rate were 40% (4/10 patients) and 78% (7/9 patients), respectively. For MMR-proficient colorectal cancer the iORR and 20-weeks PFS were 0% (0/18 patients) and 11% (2/18 patients), respectively. Retrospective expansion to 149 patients with 15 different tumor types confirmed an ORR of 39.6%, with a 7% complete RR [2] for MMR-d tumors. Moreover, a follow up phase 3, randomized trial among 307 patients with metastatic MSI-H–MMR-d colorectal cancer showed that pembrolizumab was superior to chemotherapy with respect to PFS 16.5 vs. 8.2 months) [3].

Because patients with MMR-d tumors respond to pembrolizumab, it is plausible that tumors with other types of DNA repair deficiency, such as homologous recombination (HR) repair, might be susceptible to immune checkpoint blockade.

**Fig. 1:**
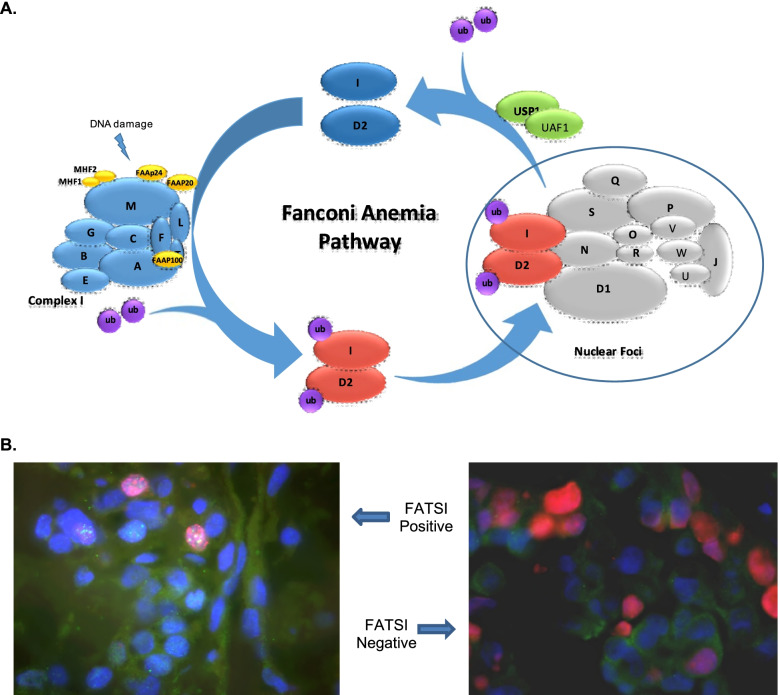
**A** The Fanconi anemia pathway and formation of repair foci. Following DNA inter-strand crosslink damage, the FANCM-FAAP24-MHF1-MHF2 anchor complex recruits the FA core complex I, which functions to activate FANCD2 and FANCI by mono-ubiquitinating the proteins. The activated FANCD2 and FANCI heterodimers are subsequently transported to subnuclear foci (encircled), which in collaboration with additional genes result in homologous recombination DNA repair. **B** The Fanconi Anemia triple stain immunofluorescence method (FATSI) was performed in Paraffin Embedded Solid Tumor slides stained with FATSI as observed with immunofluorescence microscope (400x). Left, FATSI positive (competent pathway); Right, FATSI negative (deficient pathway). DAPI (blue), Ki67 (red) and FANCD2 (green)

The BRCA genes have been identified as inherited cancer predisposition genes, as well as potential predictors of response to PARP inhibitors [4–8]. They interact with several others in the Fanconi Anemia (FA) HR pathway [9–18]. Seventeen complementation groups/genes plus other interactive proteins have been described. Monoubiquitination of FancD2 and FancI by an FA core complex followed by nuclear co-localization with other DNA damage response proteins results in the formation of nuclear repair foci; thus foci formation is the focal functional output of this pathway (Fig. 1). Based on the functional understanding of the pathway, we developed an immunofluorescence-based method, FancD2/DAPI/Ki67 (Fanconi Anemia Triple Stain Immunofluorescence - FATSI), which permits the observation of FancD2 foci formation (or lack thereof) in the nucleus of proliferating cells in paraffin embedded tumor tissues (Fig. 1) [19].

In a previously reported clinical trial, we consented 724 patients with a wide variety of solid tumors for FA foci formation screening [20]. Functional deficiency was observed in 28% of solid tumor patients tested. Subsequently, 61 treatment refractory patients identified as FA deficient per FATSI were treated with veliparib or veliparib combined with mitomycin C. Six prolonged antitumor responses occurred. PBMC BRCA analyses (Myriad Genetics, Salt Lake City, UT) were performed in 51 patients showing five patients to be carriers of BRCA-deleterious mutations. Moreover, a targeted FA sequencing panel performed in 49 FATSI negative specimens from 29 random patients identified 34 unique alterations. Alterations of note included BRCA tumor mutations with high VAF and demonstrated loss of heterozygosity in two of the germline BRCA carriers; a RAD51c (c223_224insA p.Y75*) with high VAF in a breast cancer patient experiencing a long duration antitumor response which was also detected in subsequent germline testing (Invitae, San Francisco CA); and both an ATM c.6976–1 G > T, not present in the germline, and an ERCC4 missense mutation (P379S) in both germline and tumor, in a lung carcinoma patient with tracheal infiltration experiencing massive hemoptysis after his first and only cycle of veliparib [20]. Tumors and adjacent tissue from 10 patients FATSI-positive per screening were analyzed as controls with the FA sequencing panel. A deleterious mutation (ERCC4), along with a germline potentially damaging mutation in FAN1, was found in only one patient.

We hypothesized that FATSI staining, given its ability to differentiate between functionally deficient and functionally competent FA pathway tumors, could identify additional patients susceptible to pembrolizumab for which no FDA approved indications exist. Rather than patient pre-selection, a design that incorporates all comers with a post-hoc blinded tumor FATSI analysis approach was considered more suitable for preliminary evaluation of this concept.

## Methods

The Institutional Review Boards of Baptist Health South Florida and Western IRB approved this study (*clinicaltrials.gov*- *NCT03274661*). Patients (age > 18 years) with metastatic or recurrent solid malignancy who had progressed on first line standard of care treatment or for whom defined standard of care does not exist, and for whom there was not an FDA approved indication for pembrolizumab were offered participation in the trial.

Other eligibility requirements included progressive disease, measurable as per RECIST 1.1 criteria [21], and a lapse of 4 weeks from chemotherapy or radiation therapy. Patients needed an ECOG performance status ≤2 and normal organ and marrow function [absolute neutrophil count ≥1.5 × 10^9^; platelets ≥100 × 10^9^; hemoglobin ≥9 g/dL; serum creatinine and bilirubin ≤1.5 x upper limit of normal (ULN); AST/ALT ≤2.5 x ULN]. Exclusions requirements comprised pregnancy, active brain metastases or carcinomatous meningitis, active autoimmune disease that required systemic treatment within the past 2 years, uncontrolled concurrent illness, interstitial lung disease, diagnosis of immunodeficiency, receiving systemic steroid therapy or other form of immunosuppressive therapy within 7 days prior to the first dose of trial treatment, previous treatment with immune checkpoint inhibitors, active hepatitis or tuberculosis, or having received a live vaccine within 30 days. Patients with known MMR-d cancer (i.e., with microsatellite instability, MSI-H) were excluded, as they could receive pembrolizumab as per standard of care.

### Treatment plan

Patients who met eligibility criteria and signed informed consent received pembrolizumab 200 mg as a 30-minute intravenous infusion on day 1 of every 3 weeks cycles. Pembrolizumab was provided in 50 mg lyophilized powder for injection or 100 mg in 4 mL solution for injection from Merck & Co., Inc. (Kenilworth, NJ) as an investigational product. Withholding or discontinuation of pembrolizumab followed recommendations as per pembrolizumab (Keytruda®) prescribing information.

### Tumor imaging and assessment of disease response and toxicity

Tumor assessments were performed by computer tomography (CT) or Magnetic Resonance Imaging (MRI). Measurable disease on scans obtained within 21 days of first dose of therapy was required. Scans were repeated every 2 cycles (6 weeks) and Immune-related Response Criteria (irRC) [22] were utilized for assessment of response to therapy. The Common Terminology Criteria for Adverse Events (CTCAE) version 5 was utilized for the grading of toxicities (https://ctep.cancer.gov/protocoldevelopment/electronic_applications/docs/CTCAE_v5_Quick_Reference).

### Biomarker and correlative studies

Archival paraffin embedded tumor tissue of patients participating in the trial was retrieved and sent to the Department of Pathology at Baptist Hospital of Miami. Tissue sections were cut to 4 μm and analyzed by FATSI staining, as previously described [19, 20] to assess for FA functional deficiency.

Given reported preclinical data associating certain microbiota with anti-tumor response to immune checkpoint inhibitors [23–25], we incorporated collection of stools samples in this trial from agreeing patients. For microbiome analyses, self-collection stools samples in Zymo tubes were solicited from consented patients at screening, week 7 and at the end of trial and kept refrigerated for batch analyses. Samples were shipped to Translational Genomics (TGen), Flagstaff, AZ, where DNA was extracted using the KingFisher MagMAX microbiome Ultra Nucleic Acid Isolation Kit (ThermoFisher). Bacterial DNA was quantitated by BactQuant assay [26]. Whole metagenome libraries were constructed using the KAPA HyperPrep Kit. Libraries were sequenced on an Illumina NextSeq (2 × 150 bp) instrument.

### Statistical analysis

The primary endpoint was iORR. We expected that similar to MMR-d patients, the iORR will be ≥40% in patients with functional FA deficiency (FATSI-Negative) and < 10% in patients without either HR repair deficiency or MMR deficiency. Based on our prior screening data, we anticipated that 25 to 30% of patients with solid tumors will be FA functionally deficient. We utilized a two-stage phase II design to detect an iORR of ≥20% in the whole population tested (which will include FATSI positive and negative patients) vs. the null hypothesis that the true iORR is ≤5%, representing a response by chance alone, or other infrequent unknown mechanism. H0: iORR ≤5% vs. H1: iORR ≥20% with 90% power and a Type I error rate of 10%. The alternative hypothesis of 20% iORR represented a weighted average of the anticipated 40% response in FATSI-negative patients and 10% response in FATSI-positive, assuming a 3 to 7 ratio of these patient groups. Interim analysis required that at least two of the first 20 evaluable patients enrolled had a response. If this occurred, 19 additional evaluable patients were to be accrued for a total of 39. Overall rejection criterion of the null hypothesis was observing at least four responses. The proposed two-stage design was chosen instead of the Simon Optimal or Minimax because it has a larger first stage enrollment and thus a higher expected number of FATSI-negative patients in the interim analysis. The 90% confidence interval estimates of iORR both overall and by FATSI status using the exact method (Clopper-Pearson) was calculated. We noted, however, that the planned study was small for a well-powered comparison given that we expected only 12 FATSI-negative patients. Instead, variation in iORR by FATSI status was assessed by considering the one-sided 95% lower confidence limit for the difference. Secondary endpoints included median iPFS and 20-week iPFS. In addition, we conducted a logistic regression analysis to find the association between FATSI status and iORR with adjustment for possible confounders, such as age, sex, race and number of prior treatment regimens. We used Firth method to account for sparse data bias.

The evaluation of the microbiome in stool samples was to derive clusters of patients with distinct microbiomes. Association to clinical endpoints was documented (but no formal statistics assessed) to serve as hypothesis generating for future studies.

## Results

### Patients characteristics

From November 2017 to November 2018, 41 (39 evaluable) patients were enrolled at Miami Cancer Institute clinics to fulfill the two-stage design. The characteristics of the enrolled patients are depicted on Table 1. The majority of patients [27] had gynecological malignancies, although patients with other malignancies with no FDA approval indications for pembrolizumab were also enrolled. Twenty-three (56%) of the patients enrolled were Hispanic/Latinos, reflecting the Miami-Dade County population demographics. The median number of prior systemic therapy regimens was two (range 1 to 7). The study was amended in February 2019 to allow an expansion cohort (11 additional patients) with MSI stable endometrial cancer (MS-EC).

**Table 1:** Patients characteristics

Enrolled patients, No. (evaluable)	41 (39)
Median Age, Y (Range)	62 (36–83)
Sex	34 F, 7 M
Race/ethnicity	14 W, 3 AA, 1 A, 23 H^a^
Median No. of prior regimens (range)	2 (1–7)
Primary diagnosis	
Ovarian	11
Endometrial	9 (2 carcino-sarcomas)
Pancreatic	2
Colorectal	4 (1 neuro-endocrine)
Cervical	2
Fallopian	2
Vaginal	2
Head and neck	2 (1 adenoid cystic),
Breast, esophagus, small-cell lung, small bowel, thymic, vulvar, Mullerian	1 each

^a^*W* White, *AA* African American, *A* Asian, *H* Hispanic/Latino

### Toxicities

Three hundred thirty-six cycles (range 1 to 35) of pembrolizumab were administered on trial. Pembrolizumab toxicities were consistent with previously published data (package insert). Grades 3 to 4 toxicities included nausea/vomiting (*n* = 1); abdominal pain/bowel obstruction (*n* = 2); dyspnea (*n* = 1); hyperglycemia and fatigue (*n* = 1 each). Two patients discontinued pembrolizumab due to intolerance or drug attributed toxicities (a patient with a fatal chronic obstructive pulmonary disease exacerbation during the second cycle, and a patient with grade 2 fatigue after the first cycle).

### Antitumor activity

Imaging assessments were performed every 6 weeks. One patient was deemed not response-evaluable after a further review of baseline CT images demonstrated not clearly measurable disease. Another patient deteriorated rapidly due to tumor progression within a week of the first dose of treatment. Two antitumor responses occurred among the first 20 evaluable patients, so the study continued to the planned full accrual. Among 39 evaluable patients, response evaluation showed 2CR, 5PR, 11SD, and 21PD, (iORR 18%) (Table 2). The median intent-to-treat (*n* = 41 patients) iPFS was 47 days (range: 26 to non-reached) and the 20-week iPFS was 32% (13/41).

**Table 2:** Antitumor responses according to FATSI staining

Best Response	All (*n* = 41)	FATSI + (*n* = 29)	FATSI Neg. (*n* = 10)	(ND/In, *n* = 2) ^c^
CR	2	0	2	0
PR	5	2	3	0
SD	11	9	2	0
PD	21	17	3	1
NE	2	1	–	1
iORR	18%	7%	50%	0%
MiPFS^a^ (range)	47 (26-NR)^b^ days	43 (26-NR) days	202 (41-NR) days	39 (38–40) days
20 Weeks PFS	32%	21%	70%	0%

^a^*MiPFS* Median progression free survival, ^b^*NR* Not reached, ^c^*ND/In* Not done/insufficient, *NE* Non-evaluable

### Correlative studies

FATSI analysis was performed in a blinded fashion at the end of two-stage phase 2 trial accrual. Thirty-nine tissue sections specimens from 39 patients were successfully analyzed. Two patients had either an insufficient tumor specimen (*n* = 1) or tissue sections with low Ki67 (*n* = 1). Tumor specimens from 10 patients (26%) were FATSI negative. The tumor histology distribution of the FATSI negatives were as follows: 5 endometrial carcinomas, 1 ovarian papillary serous, 1 vaginal, 1 esophageal squamous, 1 colon adenocarcinoma and 1 adenoid cystic carcinoma of the mandible. The iORR of the FATSI negative patients was 50% (95% CI, 19 to 81%) (5 of the 7 responses, including 2 CRs of long durations [11 months and > 35 months so far]) and their disease control (CR/PR + SD) rate (2CR, 3PR, 2SD) was 70%.

Twenty-nine patients (74%) had FATSI positive tumors. The iORR of the FATSI positive patients was 7% (2/29) (95% CI, 0 to 16%), and their disease control rate (2 PR, 9 SD) was 38%. Median PFS were 202 days for patients with FATSI negative tumors and 43 for FATSI positive, and the 20-weeks PFS 70 and 21%, respectively. Despite the small numbers, the differences for iORR and 20-week iPFS were statistically significant (*p* = 0.0022 and 0.0043, respectively). Table 2 depicts clinical endpoints according to FATSI tumor status.

Responding patients with FATSI negative tumors included two experiencing ≥1-year iPFS (CR and PR). They were previously treated (2–4 prior systemic regimens) patients with MS-EC. Two other MS-EC FATSI negative patients responded (CR and PR) and the fifth response occurred in ovarian papillary serous patient (PR).

Responses in patients with FATSI positive tumors (both PRs) included one patient each with small bowel carcinoma and a cervical cancer patient. Of interest, the small bowel carcinoma patient who had a PR and went on to receive 35 cycles of pembrolizumab on trial, had his tissue re-evaluated for microsatellite instability. Results showed MSI-H; thus, the patient went off trial and continued to receive pembrolizumab as per standard of care. The one other FATSI + tumor response (PR of 5 months duration) was a heavily pre-treated metastatic cervical cancer patient with high tumor mutation load as per next generation sequencing testing performed prior to enrollment.

Given the observed favorable clinical activity in enrolled MS-EC patients, 11 additional MS-EC patients, including a carcinosarcoma were enrolled. Response evaluation in this group showed 1 PR, 4SD and 6 PD. iMPFS was 52 days (range 37 to NR). FATSI staining was performed in 10 of these patients; six were FATSI negative and four FATSI positive. The one antitumor response in this group occurred in one FATSI negative tumor patient and has persisted for more than a year (16 months progression-free at last assessment).

**Fig. 2:**
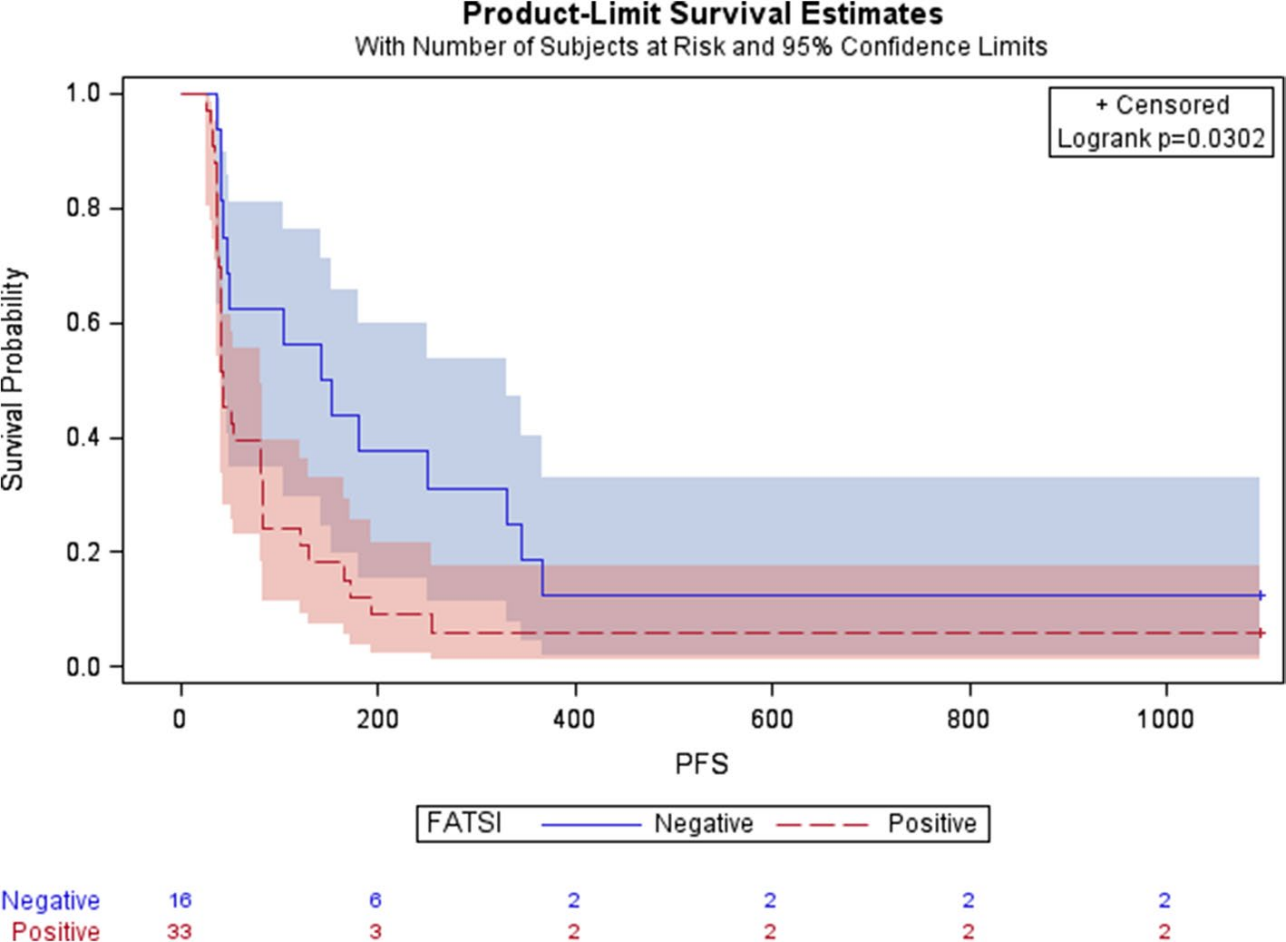
Kaplan Meier curves comparing progression free survival by FATSI status (*P* for logrank, 0.03). X-axis corresponds to progression free survival in months and Y-axis corresponds to survival probability. FATSI negative patients’ curve in blue; FATSI positives in red. + = censored

**Fig. 3:**
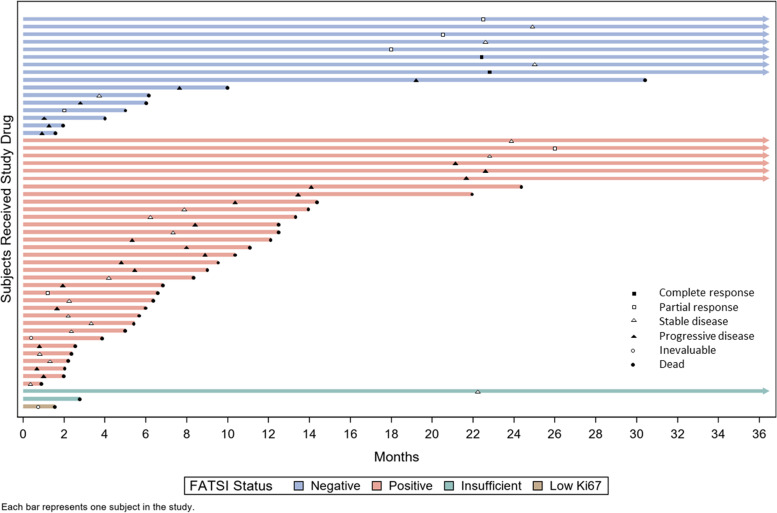
“Swimmers Plot” for Individual Patients Progression Free Survival Divided According to Their Tumors FATSI status. Each bar represents one subject on study. X-axis, number of days progression free; Y-axis individual patients. Individuals’ best tumor response outcomes are indicated by labels

Figure 2 depicts iPFS according to FATSI status for the total population of 49 evaluable patients inclusive of the expansion cohort. iPFS had not yet been reached at the data cut off of 3 years in four patients (range 16 to 36 months) (Fig. 3). Despite the small numbers of patients, considerable differences can be appreciated, favoring patients with FA pathway repair dysfunction. Logistic regression analysis showed that the adjusted odds ratio of iORR was significantly lower in FATSI positive patients (iOR, 0.144, 95% CI: 0.023–0.899) (Table 1s).

**Fig. 4:**
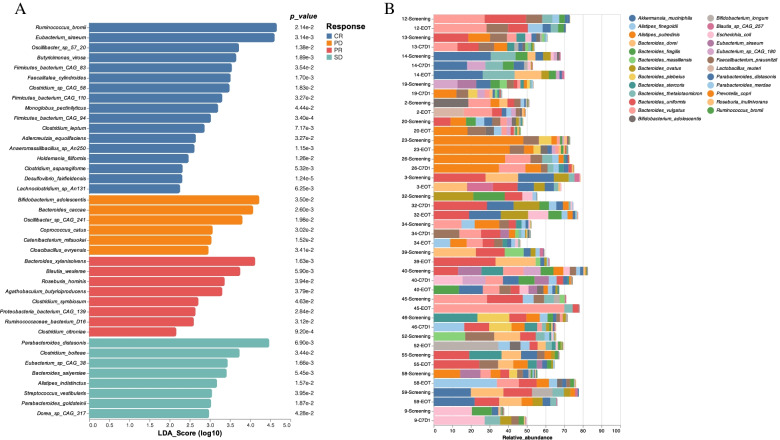
Microbiome data from stool specimens of patients on the study. **A** Taxonomy bar plots of top 25 species. **B** LEfSe plot illustrating discriminating features between response groups. CR = complete response; PD = progressive disease; PR = partial response; SD = stable disease

Forty-four stool samples from 20 patients who provided sequential samples were sequenced. Quality metrics (fastqc/multiQC) showed ≥2.3 M reads per sample. Reads were classified using MetaPhlAn3 [27], and heatmaps were generated with Seaborn 0.11.1 and hclust2. Classified reads were examined for discriminating features, in group wise comparisons using LEfSe [28]. Three hundred sixty-two species were identified in the complete dataset. Taxonomy bar plots of the top 25 species (selected by abundance) are depicted in Fig. 4A. After Bray Curtis distance hierarchical clustering (heat map not shown), discriminating features of the microbiome profile from patients experiencing tumor progression versus patients with disease stability or response (PD [*n* = 5] vs SD [*n* = 10] or PR/CR [*n* = 5]) are shown in Fig. 4B.

## Discussion

Immune checkpoint inhibition is an exciting therapeutic strategy that has revolutionized the way that solid tumor oncologists perceive cancer treatment, since a significant number of patients derive sizeable and sustained clinical benefit. Unfortunately, predictive biomarkers for clinical benefit are few and imperfect. MMR-d (per MLH1, MSH2, MSH6, and PMS2 immunohistochemistry (IHC) negative staining, or microsatellite instability assessment); PD-L1 expression; and tumor mutational burden (TMB) (when available) [1, 29] are the most common biomarkers being used in practice, with various degrees of success for patient selection. Alternative biomarkers that can identify additional patients most likely to benefit from immune checkpoint inhibition are needed. The hypothesis motivating this trial is that in addition to MMR-d other major functional DNA repair deficiencies, if properly assessed, can distinguish these patients.

A limited number of genomic NGS based panel assays have been incorporated to the assessment of HR deficiency in patients with some solid tumors such as breast, ovarian and prostate. This is based on the understanding that HR repair deficiency not only can predispose patients to cancer development, but also makes them more likely to derive clinical benefits from DNA breaking cytotoxics and PARP inhibitors [5–7, 30–33]. A large number of cancer predisposition HR mutated genes are represented in the FA pathway [9–17]; however, some of these are not routinely evaluated. Moreover, FA genes can undergo epigenetic changes that renders them functionally inactive [34, 35]. The FATSI test evaluates FANCD2 foci formation in the nucleus of proliferating cells, assessing endpoint functionality of the pathway [18], with the capability of potentially identifying germline loss of heterozygosity, as well as sporadic and epigenetic events that render HR functionally ineffective. In our hands with over 700 patients tested, 15–35% of solid tumors depending on their histological type are unable to form FANCD2 repair foci [19, 20]. It is unclear, whether there is overlap with other biomarkers, such as PD-L1 expression or TMB. Mismatch and HR repair are intrinsically linked and compensatory in normal and tumor cells. Thus, the prevailing thought is that overlap of both types of repair deficiency in the same tumor cells is unlikely [36].

The results of this study corroborate the clinical observation that some patients with advanced tumors for which there is not an FDA approved indication for single agent pembrolizumab can derive benefit from this agent. Close to a third of the patients treated had 20 weeks iPFS or longer. The FATSI analysis was performed successfully from archived tumor material in 49 patients, reflecting the simplicity of sampling preparation and analysis, as long as sufficient tumor tissue is available for slide preparation, akin to other IHC routinely performed tests. Because the test targets absence of FANCD2 nuclear repair foci (FATSI negative) to detect FA repair deficiency, it is very important to exclude false negatives, especially those in low proliferating tumors. The incorporation of Ki67 as one of the immunofluorescence test antibodies to determine sufficient tumor cell proliferation largely eliminates this caveat.

Including the expansion cohort, 16 of 49 patients tested (33%) were FATSI negative. Their clinical outcomes were better (iORR 38%, miPFS 142 days, 20 W-iPFS 56%), and predominantly drove the clinical benefit with pembrolizumab observed for the whole group. The response rate for FATSI negative endometrial cancer patients was 45% (5/11, including 2 CRs). Although, it is a smaller sample size, it is tempting to put this response rate in perspective to the 13% (3/24) response rate for pembrolizumab for PDL-1 positive endometrial cancer patients in KENOTE 028 [37].

Table 3 depicts the available results for relevant biomarkers that could serve as potential confounding factors for the clinical benefit differences observed between FATSI negative and positive tumors. These include PDL-1 staining and TMB.

**Table 3:** Tumor Histology, Best Response, and Biomarkers

Tumor Histology	FATSI	PD-L1 Score	TMB Score	Response
Ovarian (Papillary Serous)	Negative	N/A	N/A	PR
Esophagus Squamous	Negative	N/A	Intermediate	SD
Endometrial	Negative	Negative|0%	9 muts/Mb	CR
Endometrial	Negative	Negative|0%	7 muts/Mb	SD
Endometrial	Negative	N/A	N/A	PR
Adenoid Cystic Carcinoma	Negative	N/A	7 muts/Mb	PD
Endometrium	Negative	N/A	N/A	CR
Vaginal	Negative	N/A	N/A	PD
Endometrium	Negative	N/A	N/A	PR
Colon	Negative	Negative|0%	9 muts/Mb	PD
Endometrial	Negative	Negative|0%	7 muts/Mb	PD
Endometrial	Negative	N/A	5 muts/Mb	PD
Endometrial	Negative	Negative|0%	14 muts/Mb	SD
Endometrial	Negative	N/A	N/A	SD
Endometrial	Negative	CPS >/= 1	N/A	PR
Endometrial	Negative	Negative|0%	6 muts/Mb	PD
Colon	Positive	N/A	NA	PD
Thymic	Positive	N/A	Low/3.5	SD
Ovarian	Positive	N/A	N/A	PD
Ovarian	Positive	N/A	N/A	PD
Fallopian Tube	Positive	N/A	N/A	PD
Cervical Squamous	Positive	N/A	High	PR
Pancreatic	Positive	N/A	N/A	PD
Endometrial Carcinosarcoma	Positive	Negative	Low/4	PD
Cervical Squamous	Positive	N/A	N/A	SD
Fallopian tube	Positive	N/A	N/A	SD
Pancreatic	Positive	N/A	Intermediate	PD
Endometrial	Positive	Negative	Low/6	PD
Ovarian (Serous)	Positive	Negative	Low/4	PD
Ovarian	Positive	N/A	N/A	SD
Breast	Positive	N/A	7.9 muts/Mb	SD
Ovarian (Serous)	Positive	N/A	10 muts/Mb	PD
Mullerian Remnant Papillary Serous	Positive	Negative|0%	6 muts/Mb	SD
Ovarian	Positive	N/A	N/A	NE
Neuroendocrine Colorectal	Positive	N/A	N/A	PD
Ovarian	Positive	N/A	N/A	PD
Ovarian	Positive	Negative|1+, 2%	N/A	PD
Tonsil	Positive	N/A	N/A	SD
Ileum	Positive	N/A	N/A	PR
Uterine Sarcoma	Positive	Positive | 2+ 95%	4 muts/Mb	SD
Vulvar	Positive	CPS < 1 0	4.2 muts/Mb	SD
Vaginal	Positive	N/A	N/A	PD
Ovarian	Positive	0%, Negative	4 muts/Mb	PD
Endometrial	Positive	N/A	N/A	PD
Ovarian	Positive	N/A	N/A	PD
Endometrial	Positive	N/A	N/A	SD
Endometrial	Positive	N/A	N/A	PD
Endometrial	Positive	CPS 3; 0% in older test	4 muts/Mb	PD
Endometrial Carcinosarcoma	Positive	Negative|0%	8 muts/Mb	PD

Multiple studies have reported that a favorable gut microbiome is associated with responses to ICIs, although with limited concordance among identified species [38–40]. Fecal microbiota transplant from responding melanoma patients to those resistant to ICIs resulted in reversal of resistance in some patients [41]. Significantly enriched taxa in responders included the Lachnospiraceae, Ruminococcaceae, Bifidobacteriaceae, and Coriobacteriaceae families. Similar to the cited studies, our sample size is small, although sequential sampling provided for both permanence and abundance. Of note, the patient with CR had *Ruminococcus bromii* in her stool samples. Our data, although limited, may serve to supplement larger datasets being created to continue to explore the intriguing observed interactions between immune response and the human gut bacterial commensalism.

## Conclusions

The results of our study are encouraging. However, as noted on Table 3, PDL-1 and TMB measures were not correlatives required for the study and therefore did not obtain these for every patient due to clinical and insurance coverage practices. This introduces a significant confounding factor.

However, supporting the study rationale, our results suggest that beyond genomic signatures, FA pathway functional assessment should be taken into consideration, not only to enrich for patients most likely to derive benefit from PARP inhibition treatment, but also for treatment with ICIs. Additional tumor-specific studies evaluating FATSI as an enrichment biomarker supporting treatment strategies featuring immune checkpoint inhibitors, alone or in combination with PARP inhibitors, are needed.

## Supplementary Information

**Additional file 1: Table 1S.** Association between FATSI status and iORR.

## Data Availability

Collected and reported analyzed Data exists at Miami Cancer Institute, Baptist Health South Florida, Clinical Research Department.

## Abbreviations

MSI-H: Microsatellite Instability High
HRR: Homologous-recombination-repair
FA: Fanconi Anemia
iORR: Immune Response Criteria response rate
PFS: Progression-free survival
MS-EC: MSI stable endometrial cancer
ICIs: Immune checkpoint inhibitors
F: Female
M: Male
CR: Complete Response
PR: Partial Response
SD: Stable Disease
PD: Progressive Disease
NE: Non-Evaluable
MMR-d: Mismatch repair deficient
FDA: Food and Drug Administration
ULN: Upper limit of normal
CT: Computer tomography
MRI: Magnetic resonance imaging
CTCAE: Common Terminology Criteria for Adverse Events
Q: Quartile
IQR: Interquartile range
N: Number
NR: Not reached
