# Supplementary material for: Pembrolizumab activity in patients with Fanconi anemia repair pathway competent and deficient tumors
Source: Biomark Res. 2022 Jun 3;10:39. doi: 10.1186/s40364-022-00386-0 (PMC9164357; doi:10.1186/s40364-022-00386-0)
Supplement: Supplementary file 1 — Additional file 1: Table 1S. Association between FATSI status and iORR. [file 40364_2022_386_MOESM1_ESM.docx]

Table 1S. Association between FATSI status and iORR.

| **Variables** | **Adjusted Odds Ratio (95% CI)** |
| --- | --- |
| FATSI |  |
| Negative | Reference |
| Positive | 0.144 (0.023-0.899) |
| Age | 1.006 (0.917-1.105) |
| Sex |  |
| Male | Reference |
| Female | 0.947 (0.110-8.144) |
| Race |  |
| White | Reference |
| Black | 0.603 (0.038- 9.615) |
| Hispanic | 0.701 (0.112-4.403) |
| Asian | 0.264 (0.002-28.217) |
| Number of prior regimens |  |
| 1-3 | Reference |
| 4-7 | 1.292 (0.210-7.935) |
